# Owning Decisions: AI Decision-Support and the Attributability-Gap

**DOI:** 10.1007/s11948-024-00485-1

**Published:** 2024-06-18

**Authors:** Jannik Zeiser

**Affiliations:** Leibniz Universität Hannover, Institut für Philosophie, Im Moore 21, 30167 Hannover, Germany

**Keywords:** Responsibility, Attributability, Accountability, Artificial intelligence, Agency, Machine learning, Decision-support systems

## Abstract

Artificial intelligence (AI) has long been recognised as a challenge to responsibility. Much of this discourse has been framed around robots, such as autonomous weapons or self-driving cars, where we arguably lack control over a machine’s behaviour and therefore struggle to identify an agent that can be held accountable. However, most of today’s AI is based on machine-learning technology that does not act on its own, but rather serves as a decision-support tool, automatically analysing data to help human agents make better decisions. I argue that decision-support tools pose a challenge to responsibility that goes beyond the familiar problem of finding someone to blame or punish for the behaviour of agent-like systems. Namely, they pose a problem for what we might call “*decision ownership*”: they make it difficult to identify human agents to whom we can attribute value-judgements that are reflected in decisions. Drawing on recent philosophical literature on responsibility and its various facets, I argue that this is primarily a problem of *attributability* rather than of *accountability*. This particular responsibility problem comes in different forms and degrees, most obviously when an AI provides direct *recommendations* for actions, but also, less obviously, when it provides mere *descriptive* information on the basis of which a decision is made.

## Introduction: Automated and Aided Decisions

Artificial intelligence (AI) is increasingly used to assist or automate decision-making in important domains such as medicine, human resources and law. Yet, many people maintain that there is something special about decisions based on human judgement. While research has repeatedly shown that human decisions tend to be flawed and biased, and can potentially be improved by algorithms (Kahneman et al., 2021; Kleinberg et al., 2018), people regularly prefer human over automated judgement (Burton et al., 2020; Jauernig et al., 2022). At a regulatory level, the EU’s General Data Protection Regulation (GDPR) has codified the value of human judgement in Article 22(1), stating that the “data subject shall have the right not to be subject to a decision based solely on automated processing […]” (GDPR, 2016).

There are different interpretations of what it is, exactly, that Article 22 is intended to protect, and consequently what it is that makes human decisions special. Plausible rationales for Article 22 include protecting against erroneous and potentially discriminatory decisions, preserving human responsibility, and upholding human dignity by keeping humans in decision-making loops (Bygrave, 2020, p. 526; Djeffal, 2020, pp. 853–854; Hartmann & Kriebel, 2021, p. 140). From a philosophical perspective, Binns (2022) argues that only human judgement can serve *individual justice*, such that the particularities of an individual case are respected.

These debates raise a central, but understudied question: what actually makes a decision a *human* one in an ethically relevant way? As I will argue, this question concerns human responsibility, but differently from how responsibility is usually understood regarding AI. Conventionally, responsibility for AI technology is discussed as a problem of *accountability*: since no agent has sufficient control or knowledge of an AI’s behaviour, we have difficulty finding an appropriate target for blame, praise or punishment (Matthias, 2004; Sparrow, 2007). This framing of the responsibility problem is plausible for agent-like systems that act on *behalf* of human agents. However, most AI systems today actually operate as decision-support systems (AI-DSS), or “algorithms in the loop” as Green and Chen (2019) put it.^1^ In such a setup, humans are technically the final decision-makers. Here, the challenge is to ensure that human contribution is meaningful, and not merely a “rubber-stamping” (Wagner, 2019) of quasi-automated decisions.

I address this challenge by providing an account of responsibility as *attributability*, drawing primarily on a distinction between accountability and attributability developed by Watson (1996) and other philosophers. Attributability assumes that actions express the character of an agent, and decisions reflect certain qualities that belong to that agent: a lenient judge makes lenient decisions; a careful doctor makes cautious diagnoses and treatments, and so on. More specifically, decisions reflect a decision-maker’s *value-judgements*. A decision belongs to an agent in the sense that it reflects her value-judgements, which she should also ideally be able to explain and justify, i.e., be *answerable* for (cf. Shoemaker, 2015). By examining AI-DSS from this underexplored perspective, this article contributes to increasing our understanding of what constitutes meaningful human agency in the context of using AI for decision-making. Ensuring that decisions reflect value-judgements of human decision-makers is a piece of the puzzle in efforts to create systems that are human-centric (see Bryson & Theodorou, 2019).

I proceed as follows. In sect. "The Faces of Responsibility and Their Relevance for AI", I distinguish between *attributability*, *answerability* and *accountability* as different aspects of the notion of responsibility. I show that in the discourse on responsibility and AI, *accountability* has become the dominant concern. In sect. "The Attributability-Gap", I argue that while accountability may be the primary challenge for agent-like systems, the main problem raised by decision-support systems is to properly attribute decisions. I consider general strategies for ensuring that decisions made with the help of AI are attributable to human agents. In sect. "Prescriptive Decision-Support" and "Descriptive Decision-Support", I explore in detail *how* AI-DSS can infringe on attributability. I do this by considering different ways in which decision-support systems can guide human action: (i) by providing a recommendation for a particular course of action (*prescriptive systems*); and (ii) by providing relevant information without direct reference to a particular course of action (*descriptive systems*). While it seems clear that *prescriptive* systems presuppose certain value-judgements that may differ from a human agent’s values, I argue that even *descriptive* systems, while not directly suggesting actions, can undermine human attributability.

## The Faces of Responsibility and Their Relevance for AI

‘Responsibility’ is a term that is widely used, with various meanings. It is traditionally debated in philosophy, especially as *moral* responsibility (see Talbert (2022) for an overview), as well as political science and public administration scholarship, as a way of ensuring compliance with rules and holding powerful agents accountable (Thynne & Goldring, 1981; Mulgan, 2003; Bovens, 2007; Lindberg, 2013). In this section I will briefly outline the conceptual landscape that I am concerned with in this article.^2^

Following the terminology of philosophers Watson (1996) and Shoemaker (2011, 2015), I distinguish three different ‘faces’ of responsibility: accountability, answerability and attributability. Especially the contrast between *accountability* and *attributability* is going to be of interest for my purposes.

*Accountability* refers to the practice of holding agents responsible in reacting to misconduct by assigning blame or imposing legal sanctions. As Watson (1996) points out, this practice is usually subject to certain preconditions: the agent must *know* what she is doing, and she must have *control* over her actions. Otherwise, it wouldn’t be fair to hold her accountable. Accountability is concerned with questions such as “who is to blame?” or “who is to praise?”, referring to agents’ epistemic position and causal roles.

*Attributability*, in contrast, is rooted in the idea that actions express an agent’s character. By choosing certain actions over others, we reveal our values, aims and commitments, we show what kinds of persons we are– that’s why Watson calls this view of responsibility the “self-disclosure view”.^3^ It is concerned with questions such as “whose decision was it?”, relating actions to agents’ dispositions and axiological convictions.

Finally, a*nswerability* is an agent’s ability^4^ to explain and justify their conduct to others. It has been defended as a discernible facet of responsibility by Shoemaker who takes “evaluative judgments” (Shoemaker, 2011, p. 614) to be the object of answerability, something that a moral agent can defend with reasons. Answerability explicitly focuses on “who is in a position to justify or explain a given conduct?”.

Not everyone will agree with this tripartite division of responsibility. Some may claim that there are more facets to be distinguished (e.g. Thynne & Goldring, 1981), while others may want to reduce them. The philosopher Smith (2012), for example, argues that *answerability*, i.e. being open to demands for justification of one’s conduct, is really the only kind of responsibility. Conversely, some authors in the public administration literature merge answerability and accountability: as a mechanism to constrain the exercise of power (Lindberg, 2013), accountability is commonly defined as the relationship between a *forum* (e.g. a parliament or a court) and an *actor* (e.g. a public official) where the forum can demand *a justification* from the actor and possibly impose *sanctions* (Mulgan, 2003; Bovens, 2007; Novelli et al., 2023).^5^

Certainly, these various notions have been developed in different academic traditions and for different analytical purposes; it would be misguided for one discipline to claim authority over the concept. The reason I rely on the above tripartite distinction is that each of the three facets is readily discernible in the context of AI decision-support systems, and each is interesting for different reasons. The questions “who is to blame?”, “who made the decision?” and “who is in a position justify this decision?” are clearly different questions; they may, in many common situations, have the same answer– but this is not necessarily the case. Sometimes, for example, the right person to blame didn’t actually make the decision, such as when a manager takes the blame for her employee (Bovens, 2007, p. 458). At other times, a human decision-maker may lack crucial information to provide a satisfying explanation and justification of a decision because she relied on a black-box AI system (i.e. she falls short in terms of answerability), but she may still be deserving of blame for her negligent conduct. Therefore, all three facets– even if closely related– can reasonably be distinguished, and all three are associated with responsibility in ordinary language. The above distinction of accountability, attributability and answerability thus provides analytical tools to capture a broad view of *responsibility* as a salient concept in everyday life, but also in specific settings of socioeconomic interaction or political life. In particular, I draw on it to show how AI could make decisions less attributable to human decision-makers by obscuring and outsourcing value-judgments.

Within the philosophical debate, the problem of *responsibility gaps* of AI-technology has traditionally been addressed with a primary focus on agent-like systems that act on *behalf* of human operators. In terms of the three facets of responsibility, most authors focus on *accountability* for these systems. That is, who would be a legitimate target for reactive attitudes or legal sanctions if, for example, an autonomous weapon kills civilians or a self-driving car runs over pedestrians (Danaher, 2016; Hevelke & Nida-Rümelin, 2015; Johnson, 2015; Köhler et al., 2017; Matthias, 2004; Sparrow, 2007).^6^ In addition, some authors have recently also taken *answerability* into view, i.e. the demand for explanation and justification for an AI’s behaviour (Himmelreich, 2019; Kiener, 2022; Santoni de Sio & Mecacci, 2021; Tigard, 2021).^7^ These accounts share a common interest in appropriate moral responses to robot behaviour.

For agent-like systems, a primary focus on *accountability*– and to some extent *answerability*– is natural. After all, we don’t want to claim that actions performed by robots were *actually* carried out by humans, in the sense that every instance of robot behaviour is an exercise of human agency. Rather, we want to know who should be held *accountable* for the robot’s behaviour– and who might be able to *explain* it.

However, not all AI systems are robots in the above sense. Much of what is now being discussed under the label of AI are machine-learning based systems that support human decisions without acting on their own. Importantly for this article, AI systems can provide decision-support in multiple ways. Following terminology used in the field of Business Analytics, there are *descriptive systems*, which provide information about the present state, and *prescriptive systems* which suggest concrete courses of action (Lepenioti et al., 2020; Loi, 2021).^8^ DSS are essentially *epistemic tools* that analyse and process data for the decision-task at hand. However, the final decision remains with a human agent.

As for agential AI, responsibility problems have been raised for systems that support human agency (Bleher & Braun, 2022; Cummings, 2006; Grote & Berens, 2020; Nissenbaum, 1996). Again, the focus has been on *accountability* and, accordingly, the problem is usually framed as one of error-management: in the event of an error in the decision-process, who is deemed liable or blameworthy? For example, the black-box nature of machine-learning systems can make it difficult to challenge their outputs. As a result, users may be overburdened with the task to control for errors, since they lack the epistemic capacity to do so (Baum et al., 2022; Grote & Berens, 2020).

Similar points are made by Bleher and Braun (2022). The upshot of their analyses of DSS in the medical field is that our (individualistic) notions of responsibility are challenged by complex technological systems that cannot be meaningfully probed by human operators.^9^ Crompton (2021) also questions the appropriateness of blaming human users of DSS who may be inclined to over-rely on AI due to psychological biases.^10^

While these approaches address important issues, the challenge posed by AI-DSS is not limited to finding appropriate responses to technical errors and human failures. If we take seriously the idea that AI-DSS should augment rather than replace human agency, a key question is whether human decision-makers that utilize AI can still be seen as the authors of their decisions. Here, the notion of *attributability* offers a much more insightful perspective, as I will explore in sect. "The Attributability-Gap".

## The Attributability-Gap

As outlined above, attributability is concerned with an agent’s moral character, or “practical identity” as Watson (1996, p. 234) puts it. As such, it is precisely the kind of perspective that we take when we ask about the authorship of a decision. This question can easily be rephrased as a philosophical question about attribution: Was the decision a clear exercise of the decision-maker’s agency, reflecting their practical identity? Watson calls this view on responsibility the *aretaic perspective*, as he takes it to be concerned with a person’s “virtues and vices” (Watson, 1996, p. 231). For the purposes of this paper, I approximate this view by focusing on an agent’s *value-judgements*, which are closely related to their virtues and vices, but aren’t necessarily reducible to any traditional virtue ethical theory. Rather, value-judgements are more generally at the core of an agent’s aims, commitments and intentions (cf. Watson, 1996, p. 244).

Imagine Susan, a recruiter for a large company, who is faced with a great number of applications and can only offer the job to *one* candidate. If Susan were to go through the applications all by herself, she would judge according to what she values in a candidate, on behalf of her company. Susan strives for a gender balance and diversity in her hiring choices, but occasionally prioritises team spirit and specialist skills. She values candidates that indicate a good work ethic, but is wary of workaholics. All her assessments and value-judgements leading up to a decision are what make the decision *Susan’s*. She isn’t just the decision’s friendly face; it is *her* decision in the sense that it expresses her character as a recruiter.

However, she has to decide in a timely manner, so she uses an AI-DSS to process the applications, rank them by useful skills, and recommend promising candidates. While she has a good understanding of how the tool works, some details remain opaque and difficult to customise, such as: how exactly the AI weights a candidate’s work experience against their other skills, education or details in their resume, and how a declared commitment to gender balance and diversity are factored in.

Attributability-gaps occur when a human decision-maker uses a decision-tool that presupposes values that have a significant influence on the outcome of the decision *and* that they haven’t endorsed as their own values. If Susan follows the suggestions made by the recruitment algorithm without really grasping which underlying qualities were decisive, the resulting decision has been influenced by implicit value-judgements that Susan has not previously endorsed. In this way, the decision is less *hers* than if she would have understood and endorsed the relevant values. This is not merely a problem of Susan over-relying on the DSS’s recommendation, possibly missing errors. Rather, it poses a fundamental difference for the nature of the resulting decision. Despite being technically made by a human the decision doesn’t reflect characteristics of Susan’s judgement.

In contrast to accountability approaches that focus on problems of error-management, I propose the following positive characterisation of responsibility-enabling decision-support systems, which covers both attributability and answerability:

A decision-support tool should enable a human operator to make decisions in accordance with their goals and values (attributability), in a way that allows them to normatively justify and defend their decision (answerability).

While it is consistent with this definition that answerability and attributability are strictly speaking distinct, I take answerability to be doubly desirable: first, it ensures that an agent can provide normative reasons for their decision and thus justify it towards themselves and others. Second, it provides an epistemic guarantee that *attributability* is satisfied. In order for this to be possible, transparency is required, allowing decision-makers to have insight into the values according to which a tool operates.

There are three general strategies for ensuring attributability for decisions made with an AI. First, one could ensure that decision-makers are able to *endorse* relevant value judgements presupposed by the AI. If they can’t, then it seems implausible that the underlying values are really their own. Similarly, Zheng (2016, p. 68) identifies an “endorsement intuition” in the context of implicit bias and its influence on human behaviour: there is a moral difference between a person who would endorse the influence of implicit bias if they were aware of it, and a person who wouldn’t. The former can clearly be attributed with biased behaviour, the latter not necessarily. My endorsement-strategy for AI-DSS builds on a related intuition. A recruiter who relies on a decision-tool to suggest eligible candidates may *incidentally* value the very same features in an employee as the AI-DSS does. But incidental alignment between AI’s and human’s values isn’t satisfactory. We can only claim that the decision is an expression of the recruiter’s agency if they had actual insight into what is valuable according to the AI. Second, if the decision-maker can customise the tool’s value prerequisites to fit their preferences, this would result in a more flexible way to ensure attributability. I discuss this first and second strategy in the following section.

A third strategy could be to make sure that the AI itself simply *doesn’t* presuppose any value-judgements and leaves them completely up to a human decision-maker. One could imagine this being the case for *descriptive* systems that provide epistemic support, e.g. by classifying an object in an image, and don’t recommend courses of action. While this is generally true, there are complications for such a model that I discuss in sect. "Descriptive Decision-Support".

Extant theoretical frameworks that are supposed to prevent AI responsibility-gaps are only partly useful with regard to *attributability*-gaps. For example, the concept of Meaningful Human Control (MHC), as developed by Santoni de Sio and van den Hoven (2018), is a popular means of tackling responsibility problems in AI in the design-phase. To achieve MHC in a socio-technical system, two conditions must be met: (1) the “tracking condition” demands that the system should track relevant moral *reasons*, and (2) the “tracing condition” necessitates the presence of a human along the chain who understands the system sufficiently and is willing to take responsibility for its performance.

In this sense, MHC addresses attributability concerns: the system must be aligned with relevant moral reasons, and human operators need to understand it in order to be candidates for responsibility. Veluwenkamp (2022) highlights the ambiguity between *motivating reasons*, which are agent-relative, and agent-neutral *normative reasons*. The MHC framework does not clearly specify which of these a system should track. While MHC is useful for promoting responsible AI that conforms to universal moral norms and accountable AI by identifying accountable agents, it does not specifically focus on attributability, which emphasises the agency of decision-makers and their contribution through value-judgements.^11^

It should be noted that accountability and attributability are not entirely independent perspectives. Some philosophers discussing accountability for AI-DSS implicitly assume some form of attributability to hold. For example, Crompton (2021) points out that decision-support might make it hard to identify purely human “decision-points”. Baum et al. (2022, p. 16) also present problems of properly attributing decisions, which undermines the conditions for blaming human operators. However, as these authors are primarily concerned with accountability, their discussions focus on issues of fault-allocation instead of identifying attributability as a perspective in its own right. Despite the interconnectedness of the perspectives, attributability serves a distinct purpose in clarifying issues of authenticity and ownership of decisions.

## Prescriptive Decision-Support

So far, I have argued that if we want to ensure that a human decision-maker is substantively responsible for a decision made with the help of an AI, we should care not only whether they are *accountable*, but also whether the decision is *attributable* to them, and whether they can *answer* for it. Now, the extent to which an AI-DSS supports or detracts from attributability and answerability depends crucially on what role the AI-DSS plays in practical reasoning and decision-making. This, in turn, depends on what the AI’s output is and how it is being used within the decision-process. In a straightforward case, an AI provides *recommendations* for a particular course of action. In this way, it provides a direct answer to the question *“What should I do*?” This was the case in the example of Susan’s recruitment DSS that, in some form or other, is increasingly being developed and implemented by companies (Dastin, 2018; Köchling & Wehner, 2020).

One approach for data-driven algorithms to generate recommendations would be to model other people’s past decisions and use them to recommend future decisions. An example for this are therapy prediction algorithms as described by Yang et al. (2017). Here, an artificial neural network is used to predict clinicians’ decisions for therapeutic inventions in breast cancer patients. The prediction is based on a patient’s profile as well as past decisions by other physicians. A probability score is calculated for each possible decision: “From the viewpoint of the physicians, these probabilities can be interpreted as recommendation scores.” (Yang et al., 2017, p. 46). These recommendations are therefore based on what other physicians have considered to be the right decision in past cases. The normative act of making value-judgements is thereby changed into the descriptive endeavour of aggregating expert judgements from data (Jiang et al., 2021). In such an arrangement, recommendations are generated by modelling past decisions and judgements. Of course, this is not the only way to generate recommendations; algorithm designers might simply encode recommendations themselves, so that for every prediction of the AI model there is a suggested course of action.

A prescriptive algorithm potentially has the greatest influence on the decision in terms of contributing value-judgements. It presupposes the aim of the decision-task, the means that are available and appropriate to achieve the aim, and also a particular way of weighing costs and side-effects. In some cases, at least some of these presuppositions can be safely regarded as common-sense for a given field. In medicine, for example, it seems clear that the recovery and health of patients is the overarching aim. However, the choice of appropriate means and the acceptability of side-effects may already be more debatable, and in end-of-life scenarios even the aim of therapy will not be uncontroversial. Basically, more detailed intentions of individual decisions can be difficult to generalise.

Following the first strategy for attributability-enabling AI, an operator could be given the opportunity to *endorse* value-judgements implicit in the system. An immediate obstacle in this regard is an AI’s opacity that inhibits a user’s epistemic access to its inner workings (Burrell, 2016). On a technological level, this would require explainable AI (XAI), or the use of inherently interpretable systems (Rudin, 2019).^12^ Common methods for explaining black-box AI systems can help users understand why a system produced a certain outcome: for example, counterfactual explanations tell us which changes in the input lead to changes in the output of a model (Wachter et al., 2017; Guidotti, 2022). For computer vision tasks, there are various methods for making the output of a system understandable, e.g. via heat maps (Buhrmester et al., 2021).

Baum et al. (2022) explore how XAI can help facilitate human responsibility. They argue that if we want to hold an agent accountable for an AI-assisted decision, that agent must have had access to the motivating reasons behind the AI’s output. This would require a user, like Susan the recruiter, to know which information about a candidate played a role in “determining the output of a system” (Baum et al., 2022, p. 11): e.g. what kind of work experience was considered relevant; what kind of educational history counted in a candidate’s favour; what inferences about a candidate’s psychology were taken into account.

Explainability methods can help an agent recognise when the system is operating according to different values (i.e. taking different reasons as motivating) and when it may have gotten facts wrong. Baum et al. (2022, p. 22) call these two cases “disagreement of relation” and “disagreement of fact” respectively. While both are important for responsibility overall, I take value disagreements to be an especially pressing problem for attributability. A system may be accurate in the sense of getting all the facts right, but still distort a decision-maker’s agency by *evaluating* the facts differently. In both cases, the agent is potentially hindered from realising her intentions. But in the case of a value disagreement the AI “stands for” a different normative outlook than its user. The AI is not simply descriptively wrong; it promotes different values. To allow an agent to endorse an AI’s value-judgements, both local and global XAI methods are potentially useful (Guidotti et al., 2019, p. 93:6): when picking up an AI-DSS, a user benefits from a *general* assessment of the model’s values; and for each output, she may need to endorse more *concrete* value-judgements. We should be careful, however, not to invest too much hope in current XAI methods: as Ghassemi et al. (2021, p. e748) point out with regard to heat maps: “[…] the important question for users […] is not where the model was looking but instead whether it was reasonable that the model was looking in this region.” That is, while such explanations are helpful, they are imperfect, and any proper explanation in terms of *reasons* requires an interpretation that users have to fill in. Values inherent in the recommendation of an AI-DSS won’t be made fully transparent with these methods. Therefore, a better solution might be, where possible, to use systems that are transparent and interpretable for users from the outset (see Rudin et al., 2022).^13^

However, both explainability and interpretability won’t help much if it turns out that the AI’s values are in fact different from its user’s. Here, we can turn to a *second* strategy: calibrating the AI to match the values of the decision-maker.^14^ In this sense, McDougall (2019) highlights the need for a value-flexible AI in healthcare contexts, so that patients’ autonomy is respected and the decision for a treatment according to their own values is warranted. McDougall even argues that the introduction of AI into medical decision-making could provide an opportunity for non-paternalistic shared decision-making. But while McDougall emphasises the threats to and options for patients’ autonomy, my focus here is on preserving the agency of decision-makers. In the case of medical AI, both issues may turn out to be two sides of the same coin: ideally, a physician respects a patient’s values and goals and helps them to arrive at a suitably informed decision. But in general, they are distinct concerns.

The project of v*alue-sensitive design*, for example, explicitly aims to construct technology that embodies desirable values (Friedman, 1996; Friedman et al., 2013; van den Hoven, 2007). However, as McDougall notes, *value-sensitive design* is usually concerned with generally shared stakeholder values, not so much with individual decision-makers’ differing values (McDougall, 2019, p. 3). To ensure that decision-support systems align with a specific decision-maker’s values, a more individual approach is needed. Gal (2017, pp. 66–70) presents a taxonomy of different “algorithmic assistants”, ranging from least to most paternalistic versions: “Stated Preference Algorithms” simply execute users’ choices; “Menu of Preferences Algorithms” let users select their preferences; “Predicted Preferences Algorithms” are supposed to predict a user’s preferences by analysing their data and creating profiles. Within this framework, both attributability and answerability would be best served with “stated” or “selected” preferences– where “preferences” would correspond to preferences of values.

Applying Gal’s taxonomy, one could approximately state that the more paternalistic an algorithm is, the more difficult it is for human users to identify– and potentially endorse– the relevant values at play in a decision. For example, when a therapy predictor recommends a treatment based on what other physicians have decided in the past, it is difficult for decision-makers to know *why* the system recommends a treatment. The *why* here is not just a question of which input feature had the biggest effect on the output. It’s a question of what is normatively relevant in the input that *justifies* the recommendation. Consequently, it is hard to answer what exactly the value-judgements are that determine a good therapy according to the AI. For decision-makers to be able to endorse relevant value-judgements– end thereby ensure attributability–, they would need to know how central concepts like these are operationalised by the tool.

## Descriptive Decision-Support

Clearly, systems that *recommend* courses of action have to presuppose value-judgements. But that’s not how all decision-support systems work. A third strategy for preserving human value-judgements in a decision-process might therefore be to rely on AI-DSS that simply provide evidence for humans to base a decision on, without direct reference to future courses of action. In this way, value-judgements about how to use this information in practical reasoning are left to a human agent. I call this use of AI in decision-support *descriptive*. Indeed, descriptive systems can mitigate many obvious attributability issues described above. Subtler problems may persist, however, since descriptive AI systems also don’t provide neutral, value-free information. On the contrary, biases in favour of certain goals and values may run counter to a human decision-maker’s values and goals, and thus present a problem for attributability.

An AI that provides *descriptive information* without recommending a course of action can still play a crucial role in decision-making.^15^ Take, for instance, machine-learning models in medicine that can identify diseases and aiding diagnoses. Image recognition tools have been shown to be competent, in some cases even outperforming human clinicians in their ability to detect diseases on medical images (see Liu et al., 2019 for an overview). Similarly, risk-assessment tools for forensic use, such as COMPAS, do not explicitly recommend actions either, but rather provide risk-scores for future reoffending (Angwin et al., 2016; Dieterich, 2016). In hiring processes, too, AI can be helpful not only by providing straight-out recommendations, but rather by establishing psychological profiles of candidates (Harlan & Schnuck, 2021).

Given the aims and values of a decision-maker, such information could be used to justify a number of different courses of action. Suppose a medical ML model classifies a mole as being “potentially cancerous”. Assuming that we come to believe that this output is accurate, we may consider different options: remove the mole; decide to keep an eye on it for now; or forego further treatment. Deciding on which treatment or care to provide, given individual circumstances, requires difficult value-judgements.

A human agent who reads an AI’s output as a recommendation for a decision outsources an all-things-considered verdict to the AI. Whereas an agent who applies an AI in the classificatory sense uses it to establish the truth of a decision-relevant proposition *p*– like “mole is cancerous”. Given the probability of *p*, it is the agent’s task to decide for a course of action *–* like “develop treatment plan *H”*. The role of the AI’s output, in this case, is to serve as evidence for or against *p* on the basis of which the agent forms a belief that in turn serves as the basis for a decision.

The division of labour in this arrangement seems clear: the AI is an epistemic tool that helps the human agent to form decision-relevant beliefs about the world; the human agent uses these facts in practical deliberation and, in combination with their aims and values, devises a final decision for action. One might think, then, that as long as the AI tool only serves descriptive purposes, it is entirely up to the human to express their moral character in action.

However, there are complications to this simplistic account. As has been repeatedly stressed in modern philosophy of science (Douglas, 2000; Rudner, 1953), the supposedly purely epistemic task of providing evidence for some proposition *p* usually involves non-epistemic value-judgments. In this sense, Biddle (2020) demonstrates how the process from development to deployment of algorithms is influenced by non-epistemic values that will later influence the performance of those algorithms. Even in early development stages of a machine-learning model, important value choices are involved: what exactly is the target phenomenon to be represented, and how should it be represented? How will the data be sampled, structured and pre-processed? Should the output conform to some standard of *fairness* (Verma & Rubin, 2018)?

In an influential study, Buolamwini and Gebru (2018) demonstrate that commercial gender classification-systems tend to misclassify faces of people with darker skin tones significantly more often compared to faces with lighter skin, perform worse on female faces than on male faces, and worst on darker female faces (Buolamwini & Gebru, 2018, p. 8). These disparate results are largely due to misrepresentative training data, which, by themselves, do not appear to rest on deliberate value-judgements, but rather on carelessness in the face of harmful racial bias. However, as Biddle rightly points out, there is no striving for a better practice which rests on some value-*neutral* way to organise data and program algorithms. Instead, any such attempt will have to consider issues such as historical wrongs, present prejudice, or a political standard of human diversity, to conceptualise them, quantify them accordingly, and thus settle on one of innumerable ways to structure data: “These decisions […] reflect values, including values embedded in the practical contexts in which the system will be used.” (Biddle, 2020, p. 6).

Of course, the thesis that technology, or artefacts in general, are value-laden is neither new nor exclusive to AI (Kroes & Verbeek, 2014; Winner, 1980). However, the specific question here is whether an agent who uses such artefacts in their action-planning gets disconnected from the resulting action if they cannot endorse the values implicit in the artefact: that is, whether such an agent may in some sense be alienated from their decision, as they cannot *be attributed with* or *answer for* the value-judgements that underlie the empirical premises of their action. For it seems at least plausible that value-judgements underlying our empirical practices can be significant expressions of our agency, no less than value-judgements concerning our aims and purposes.

When I make important decisions that possibly affect others, it says a lot about my personality how I conduct myself epistemically: how sure do I aim to be about the facts that underlie my decision? How careful am I to ensure that my decision does not disadvantage marginalised groups? How thoroughly have I collected data that support my decision-relevant beliefs? By relying on AI tools in decision-making, I am willing to outsource at least some of these epistemic attitudes and practices to others, and the values and qualities that these attitudes and practices represent are not necessarily my own. In particular, the inscrutability of the technology and its design processes may make it difficult for users of the technology to know whether the values embedded in the AI are truly aligned with their own.

Is this a bad thing? In general, outsourcing at least some amount of one’s epistemic practices and associated values is practically unavoidable, even apart from the use of AI-powered decision-support tools. In many fields, we have to rely on findings of scientific studies, thus ‘outsource’ the epistemic practices behind these findings to the scientists conducting the studies. In other contexts, we must rely on someone’s testimony, which may as well depend on certain value-judgements. However, AI technology may pose problems that aren’t present in these other forms of shared epistemic labour: the machine-learning pipeline is a process involving value-judgements at multiple steps (Biddle, 2020; Fazelpour & Danks, 2021) that are not always methodically formalised, but rely on intuitive judgements by algorithm designers. Domingos (2012) compares practices like feature-selection in machine-learning to a “dark art” that is “difficult to find in textbooks”.

So while explicit value-judgements regarding the choice of a preferred course of action are left to the human user, there are subtle value-judgements at play even with descriptive systems. A judge who is lenient and fair, or a clinician who diagnoses carefully and thoroughly, can only express these attributes if they understand how the tools that they use interact with these qualities. There is, of course, a limit to what an individual decision-maker can know about the tool she is using. Corresponding to the level of understanding that an agent has about a decision-support tool, attributability might not be a binary property, but a gradual one. Therefore, by using AI-DSS, users can be more or less responsible, depending on the extent to which users unknowingly outsource value-judgements, and how well their own values are aligned with the AI’s. Moreover, whether such responsibility-gaps are *problematic* depends on the importance of individual agency for a given decision task, and the significance of particular value-judgements for it. I have given examples of situations where attributability-gaps might occur, but a detailed analysis of their ethical impact is beyond the scope of this article.

## Conclusion

I have argued that algorithmic decision-support systems pose a challenge to responsibility that goes beyond what is typically discussed with regard to AI. Insofar as we want AI-DSS to preserve human authorship in decisions, we should care about decisions being attributable. That is, the relevant value-judgements expressed in a decision should reflect value-judgements of the human decision-maker who made the decision. This form of responsibility is threatened by AI-DSS because decisions made with the help of this technology can reflect value-judgements that are implicit in the technology itself, without necessarily being attributable to the human agent using the AI.

I have described several strategies for preserving attributability. First, we could give humans the chance to *endorse* relevant value-judgements implicit in the technology. In order for this to be possible, the AI’s values would have to be transparent enough to allow decision-makers to understand them. Second, we could simply leave it up to human agents to make value-judgements and reserve an informative role for the AI. I have argued that this strategy misses how descriptive AI may already presuppose certain values. And third, we might promote value-flexible systems that are adjustable to a decision-maker’s preferences. The extent to which these strategies are suitable will depend on the circumstances of each case. It is for future work to further explore how responsibility-as-attributability can be reflected in AI regulations and guidelines if the concept is supposed to be not just an analytical perspective, but a concrete policy objective for keeping humans at the centre of AI supported decisions.

It also remains to be analysed for which kinds of decisions we *want* this kind of substantive human responsibility for decisions. Where we care primarily about having structures for blame and liability in place, we could still aim for responsibility without attributability, for example through “blank check responsibility” (Champagne & Tonkens, 2015). However, where attributability is ensured, it provides a more robust basis for accountability-practices while retaining an intuitively plausible sense of human authorship in decisions.

## Footnotes

1 To be sure, even if such AI systems *support human* decisions, they themselves still “make decisions” in a technical sense: they *decide* whether to classify an image as a dog or a wolf, they *decide* on the risk score of a criminal defendant. In conventional computer science terminology, such agent-like capacities are exactly what characterises AI systems: the ability to perceive contexts and choose the best action or output according to a performance measure and learn new contexts and patterns (see Bryson & Winfield, 2017; Russell & Norvig, 2022, p. 58). However, when I speak of “decisions” in this paper, I refer to the final human decision that was supported by an AI system. I thank an anonymous reviewer whose comment led to this clarification.

2 I am grateful to an anonymous reviewer for suggesting some of the following literature, which contributes to a more comprehensive view on the concepts discussed.

3 For an overview, see Talbert (2022, section 3.1.2). Talbert distinguishes between *attributability* (a view represented by Watson) and *attributionist views.* I base my discussion on the former notion.

4 Some may prefer to characterise answerability as an agent’s *duty.* I agree that it may indeed sometimes amount to a duty, but I will treat it less demandingly as an *ability* here.

5 In the public administration literature, the concept of *attributability* has, to my knowledge, not explicitly been discussed. Rather, what I call “attributability” here could in this tradition approximately be categorised as personal moral responsibility: a discretionary commitment to one’s own values– as opposed to accountability/answerability which always involve external social control (see Mulgan, 2003, p. 15; Novelli et al., 2023).

6 Although, as Nyholm (2018) argues, we should not downplay human agency. There might be room for a *collaborative* agency between humans and robots.

7 Notably, Tigard (2021, pp. 602–603) also discusses attributability, criticising the focus on accountability in the general debate. He finds that machines do not have cares or commitments, so it would be incoherent to attribute a machine's behaviour to any underlying "character". While I agree, I focus here on how we can ensure that a decision made with the help of AI-DSS reflects the values and commitments of human decision-makers.

8 Models that make *predictions* about future states are subsumed under the *descriptive* category here. DSS aren’t necessarily powered by machine-learning or other AI algorithms. However, such data-driven approaches are likely to increase in importance (see Lepenioti et al. (2020, pp. 66–67), and my argument is tailored towards systems based on machine-learning technology.

9 This complacency may also be a result of users falsely perceiving the technology to be a moral agent of its own. Friedman and Kahn (1992) have pointed this out in an early contribution to the debate.

10 To some extent, an understanding of responsibility as *accountability* is reflected in legal documents relevant for AI regulations, such as the GDPR. For example, Article 5(2) regulates the accountability of data controllers to comply with the regulation, as does Article 24. The term is generally often used to refer to mechanisms that ensure compliance with regulations and ‘ethical’ conduct (see also Theodorou & Dignum, 2020). Novelli et al. (2023,) point out that definitions of ‘accountability’ in key European documents are quite broad. However, as stated in the introduction section of this paper, Article 22(1) GDPR can be interpreted not only as aiming at *accountability*, but also *attributability*, as it demands human involvement in important decisions.

11 This doesn’t mean that there is always just *one* individual decision-making agent involved. Shared decision-making makes attributability more complicated, but as long as human agents can agree on shared values, we can attribute decisions and actions to multiple or collective agents, too.

12 While it is well-established that making AI systems transparent is conducive to *accountability* (see IEEE 7001 Standard for Transparency of Autonomous systems, sect. 1.2), I am here exploring its relation to *attributability* as another facet of responsibility.

13 However, even interpretable models might still suffer from some of the problems described in sect. "Descriptive Decision-Support".

14 Such ideas have been explored for other kinds of AI systems, for example an “Ethical Knob” for self-driving cars to be ethically customised by users (Contissa et al., 2017).

15 Again, “decision” refers to a human decision here. Arguably, the output of such an AI tool (“cancerous/non-cancerous mole”) is a *decision* in its own right that presupposes certain goals and values. This is consistent with the idea that the AI system serves as a decision-support tool– as long as there is a final decision left to a human agent.
